# Design of an electrospun tubular construct combining a mechanical and biological approach to improve tendon repair

**DOI:** 10.1007/s10856-022-06673-4

**Published:** 2022-05-31

**Authors:** N. Pien, Y. Van de Maele, L. Parmentier, M. Meeremans, A. Mignon, C. De Schauwer, I. Peeters, L. De Wilde, A. Martens, D. Mantovani, S. Van Vlierberghe, P. Dubruel

**Affiliations:** 1 Polymer Chemistry & Biomaterials Research Group, Centre of Macromolecular Chemistry (CMaC), Ghent University, Krijgslaan 281 S4-bis, 9000 Ghent, Belgium; 2 Laboratory for Biomaterials and Bioengineering, Department of Min-Met-Materials Engineering & Regenerative Medicine, CHU de Quebec Research Center, Laval University, 2325 Rue de l’Universite, Quebec, QC G1V 0A6 Canada; 3 Faculty of Veterinary Medicine, Department of Translational Physiology, Infectiology and Public Health, Ghent University, Salisburylaan 133, 9280 Merelbeke, Belgium; 4 Smart Polymeric Biomaterials, Surface and Interface Engineered Materials, KU Leuven, Andreas Vesaliusstraat 13 - box 2600, 3000 Leuven, Belgium; 5 Faculty of Medicine and Health Sciences, Department of Human Structure and Repair, Ghent University Hospital, C. Heymanslaan 10, ingang 46, 9000 Gent, Belgium; 6 Faculty of Veterinary Medicine, Department of Large Animal Surgery, Anaesthesia and Orthopaedics, Ghent University, Salisburylaan 133, 9280 Merelbeke, Belgium

## Abstract

Hand tendon injuries represent a major clinical problem and might dramatically diminish a patient’s life quality. In this study, a targeted solution for flexor tendon repair was developed by combining a mechanical and biological approach. To this end, a novel acrylate-endcapped urethane-based polymer (AUP) was synthesized and its physico-chemical properties were characterized. Next, tubular repair constructs were developed using electrospinning of the AUP material with incorporated naproxen and hyaluronic acid (i.e. anti-inflammatory and anti-adhesion compounds, respectively), and with a tubular braid as mechanical reinforcement. Tensile testing of the repair constructs using ex vivo sheep tendons showed that the developed repair constructs fulfilled the required mechanical properties for tendon repair (i.e. minimal ultimate stress of 4 MPa), with an ultimate stress of 6.4 ± 0.6 MPa. Moreover, in vitro biological assays showed that the developed repair tubes and the incorporated bioactive components were non-cytotoxic. In addition, when equine tenocytes and mesenchymal stem cells were co-cultured with the repair tubes, an increased production of collagen and non-collagenous proteins was observed. In conclusion, this novel construct in which a mechanical approach (fulfilling the required mechanical properties) was combined with a biological approach (incorporation of bioactive compounds), shows potential as flexor tendon repair application.

**Figure:**
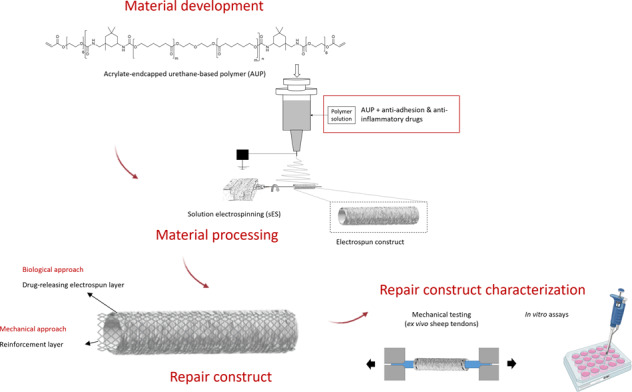
Graphical abstract

Graphical abstract

## Highlights

- Synthesis of a novel acrylate-endcapped urethane-based precursor (AUP).
- Physico-chemical characterization of the developed AUP material.
- Development of a reinforced electrospun tubular repair construct containing bioactive components.
- Mechanical evaluation of the repair constructs on ex vivo sheep tendons.
- Biological evaluation of the repair constructs by means of indirect and direct testing using mono- and co-cultures.

## Introduction

Tendon injuries are painful and persistent, and can significantly reduce quality of life for patients in which insufficient healing is observed or which were treated unsuccessfully [1]. Hand tendon lesions in particular represent one of the most frequent tendon disorders in human [2]. The hand, being the human executing body unit, is crucial during daily life, especially when considering sport activities and professional life [3]. As such, chronic overuse injuries and ruptures in the hand are frequently observed. A considerable part of trauma emergencies typically involves flexor tendon lesions of the hand, affecting one in 2700 people each year [2]. These injuries might have a significant impact on hand function and thus quality of life. Therefore, early treatment with optimal recovery is indispensable to prevent permanent dysfunction [4].

The healing capacity of tendon lesions mainly depend on the type of injury, the physical activity and age of the patient, and also on the inflammatory response following injury [5]. Whereas relatively minor tendon traumas may primarily regenerate, complicated tendon traumas, and more specifically complete ruptures or transections, result in the formation of scar tissue. The mechanical properties of this scar tissue are impeded when compared to the original tendon tissue, which often results in pain and poor functionality. Damaged tendons heal slowly, and repair often requires a long recovery period. In these cases, surgical interventions are inevitable.

Current surgical interventions rely mostly on suturing techniques or on the use of tendon grafts (i.e. autografts, allografts, xenografts, and synthetic grafts) [6–8]. The current state of the art in surgical techniques still includes suturing the injured tendon ends together, which requires a non-degenerated tendon with regeneration potential [9, 10]. Surgical repair using sutures is known to minimize scar tissue formation, increase the rate of collagenization and avoid the presence of non-tendinous tissues between the two ends of a tendon. Despite a successful surgical tendon repair, however, tendon adhesions are typically observed after tendon injury, both with the surrounding tissue and between tendon and tendon sheath [11, 12]. These adhesions emerge from the non-organized scar tissue. A second limitation of the suturing techniques is the need for a splint to (at least partially) immobilize the tendon during rehabilitation [13, 14]. This is important to protect the surgical repair and to prevent reinjury. Third, the insertion of a material during surgery often elicits a strong inflammatory response, with inflammatory cells being attracted to the injury site. In this respect, drug-releasing systems limiting adhesion and the inflammatory response have gained interest. The ability of hyaluronic acid (HA) and naproxen (Nap) to promote gliding and to counter adhesion formation around the repair complex, has been reported in literature [15].

The ideal technique of (flexor) tendon recovery should induce a healing response at the damaged tendon ends and to generate a repair site with low friction and minimal bulk, resulting in less inflammation and less adhesions (biological approach) [16]. Additionally, an optimal mechanical strength is required for tendon repair to fulfill the minimal needs of a healing tendon (i.e. mechanical approach). For an optimal repair of hand tendons, the minimal bearing stress of the construct should be approximately 4 MPa [7, 17]. Both the mechanical and biological approach are essential when developing a construct for tendon repair. However, current state-of-the-art mostly focusses on one approach, either mechanical or biological, rather than combining both [18–23]. Moreover, considering the biological approach, it is remarkable that often only one biological issue (such as adhesion) is evaluated in order to enhance tendon repair [24–27]. As such, a strong challenge in tendon repair remains in order to tackle adhesion, inflammation, and mechanical strength all together.

In the present study, a repair construct was developed representing an optimal and controlled healing environment with minimal inflammation reactions and adhesions to the surrounding tissues. To this end, both a mechanical and a biological approach was combined in a tubular electrospun repair construct. Firstly, as a mechanical approach, a novel acrylate-endcapped urethane-based crosslinkable precursor (AUP), constituting a 530 g mol^−1^ poly(ε-caprolactone) (PCL) backbone, was synthesized and characterized. To fabricate a tubular construct for tendon repair, electrospinning (ES) was elaborated as a processing technique. Subsequently, the developed repair construct was tested with regard to multiple mechanical requirements by means of tensile testing. More precisely, mechanical properties of (non)drug-loaded tubes were compared with reinforced drug-loaded tubes, both the developed constructs themselves and using ex vivo sheep tendons. Furthermore, a degradation study on the developed repair constructs was performed as well.

Secondly, in the biological approach, the proposed design aimed to provide a controlled drug release to avoid adhesion and inflammation. Therefore, both naproxen and hyaluronic acid were incorporated in order to gradually release the pharmaceutical components at the injured site. In vitro biological assays using human fibroblasts (hFBs) were assessed to test the cytocompatibility of the developed repair constructs. Moreover, to evaluate the potential supportive effect of the construct on tendon healing, collagen production was evaluated in an indirect co-culture of equine tenocytes and mesenchymal stem cells (MSCs) with the construct.

## Materials and methods

### Material synthesis and characterization

#### Material synthesis

The AUP precursors were synthesized by connecting a diisocyanate linker to a short flexible oligomer spacer (ethylene oxide, EO) to both ends of the central hydrophobic backbone polymer. In brief, the synthesis of the AUP530 material occurred through a two-step process. Firstly, the diol backbone (PCL, molar mass (MM) of 530 g·mol^−1^) reacted with isophorone diisocyanate (IPDI) in a stoichiometric 1:2 ratio. Subsequently, a radical scavenger, butylhydroxytoluene (BHT, 500 ppm), was added to the polymer solution. Next, a catalyst (bismuth neodecanoate, 300 ppm) was added very slowly. During the first step, the temperature ranged between 80–90 °C. After addition of the catalyst, the temperature remained at 75 °C. Secondly, this intermediate product (PCL-IPDI) was reacted by dropwise addition of two equivalents of the acrylated oligomeric (6 units) EO endcapping agent. Next, bismuth neodecanoate (300 ppm) was used to catalyze the reaction (again managing the temperature at 80–90 °C). The end of the reaction was identified with Fourier transform infrared (FT-IR) spectroscopy. This was carried out until complete disappearance of the absorption band of isocyanate (NCO content of IPDI) visible at 2270 cm^−1^. Next, two post-stabilizers, phenothiazine (PTZ, 500 ppm) and triphenylphosphite (TPP, 500 ppm), were added to the mixture. Finally, the reaction product was cooled down to room temperature.

#### Material characterization

##### ^1^H-NMR spectroscopy to determine the acrylate content and the molar mass

Quantitative proton nuclear magnetic resonance (^1^H-NMR) spectroscopy enabled to determine the acrylate concentration, as well as the molar mass (MM) of the PCL-diol backbone and the MM of the developed AUP material. A fixed value of 10 mg of AUP530 (*n* = 1), combined with the standard dimethyl terephthalate (DMT, 10 mg), was dissolved in 1 mL deuterated chloroform. An NMR spectrometer (Bruker Avance 400 MHz) was used to obtain the spectrum. Analysis of the obtained spectra (Fig. S1) was performed using the MestreNova software whereby a baseline correction, based on the Whittaker Smoother method, was carried out. The amount of acrylates was calculated using Equation (1). The MM of the backbone and AUP530 material were determined by Equations (2) and (3), respectively.

1 $${\rm{MM}}_{{\rm{acrylate}}\,{\rm{content}}} = \frac{{I_{\delta = 5.83 - 6.4{\rm{ppm}}}}}{{I_{\delta = 8{\rm{ppm}}}}}\times\frac{{N_{\delta = 8{\rm{ppm}}}}}{{N_{\delta = 5.83 - 6.4{\rm{ppm}}}}}\times\frac{{W_{\rm{DMT}}}}{{\rm{MW}_{\rm{DMT}}}}\times\frac{{1000}}{{W_{\rm{AUP}}}}$$

2 $${\rm{MM}}_{\rm{backbone}} = \frac{{I_{\delta = 1.4{\rm{ppm}}}}}{{N_{\delta = 1.4{\rm{ppm}}}}}\times\frac{{N_{\delta = 5.83 - 6.40{\rm{ppm}}}}}{{I_{\delta = 5.83 - 6.40{\rm{ppm}}}}}\times{\rm{MM}}_{{\rm{CL}}\,{\rm{unit}}} + {\rm{MM}}_{\rm{EO}}$$

3 $${\rm{MM}}_{\rm{AUP530}} = {\rm{MM}}_{\rm{backbone}} + 2\times\left( {{\rm{MM}}_{\rm{IPDI}} + {\rm{MM}}_{\rm{Bisomer}\,{\rm{PEO6}}}} \right)$$

with: *I*_*δ*=1.4ppm_ = integral of signal of the protons from the repeating unit of CL. *I*_*δ*=5.83-6.40ppm_ = integrals of the signal of the protons in acrylates. *I*_*δ*=8.00ppm_ = integral of the signal of the protons from the aromatic ring in DMT. *N*_*δ*=1.4ppm_ = number of protons from the repeating unit of CL (=2). *N*_*δ*=5.83-6.40ppm_ = number of protons per AUP molecule (= 6). *N*_*δ*=8.00ppm_ = number of protons in the benzene ring of DMT (=4). MM_DMT_ = 194.186 g mol^−1^; MM_CL unit_ = 114.16 g mol^−1^; MM_EO_ = 104.1 g mol^−1^; MM_IPDI_ = 222.3 g mol^−1^; MM_Bisomer PEO6_ = 336 g mol^−1^.

##### Determination of the thermal properties using thermogravimetric analysis (TGA) and differential scanning calorimetry (DSC)

Thermal stability was evaluated by TGA (TA Instruments Q50). In brief, the platinum pan was pyrolyzed with a Bunsen burner to remove residual impurities. Subsequently, 10 mg of AUP530 (*n* = 1) was loaded into the pan following a standard protocol of heating from 35 to 750 °C in an inert (N_2_) atmosphere at a rate of 10 °C min^−1^. Afterwards, a cooling equilibrium was set to 350 °C. The software (TA instruments, Universal Analysis 2000) monitored the mass loss evolution as a function of increasing temperature.

Thermal transition temperatures were recorded by DSC (Q2000, TA Instruments). An amount of 10 mg of AUP530 (*n* = 1) was placed into a T_zero_ aluminum pan with a hermetic T_zero_ aluminum lid. Within this process, equilibration was set at 45 °C and the sample was heated to 100 °C at a rate of 20 °C min^−1^, followed by cooling to −75 °C at a rate of 5 °C min^−1^. Next, the sample was again heated up to the temperature of 100 °C at a rate of 10 °C min^−1^. Afterward, data analysis was performed using the TA instruments Universal Analysis 2000 software.

##### Physico-chemical characterization of the crosslinked AUP precursor by gel fraction, swelling ratio and crosslinking efficiency determination

Circular samples (D: 16 mm) were punched out of the 1 mm thick crosslinked AUP530 sheets after 30 min of ultraviolet (UV)-crosslinking (λ = 365 nm, 10 mW cm^−1^). Every sample was first weighed in its dry state (*W*_i_) and then placed in a six-well plate, and immersed in an excessive volume of ultrapure water (to determine swelling ratio) or chloroform (to determine gel fraction). Following this, the samples were incubated at 20 °C for 72 h. After removal of the excess water or chloroform, the mass of the samples in swollen state was determined (*W*_s_). Next, samples were dried, and the mass of the dried samples was recorded (*W*_f_). The measurements were performed in sixfold (*n* = 6). The swelling ratio (SR) and gel fraction (GF) were calculated using Equations (4) and (5), respectively.

4 $${\rm{Swelling}}\,{\rm{ratio}}\,\left( {\rm{SR}} \right) = \frac{{\left( {W_{\rm{s}} - W_{\rm{f}}} \right)}}{{W_{\rm{f}}}}$$

5 $${\rm{Gel}}\,{\rm{fraction}}\left( {\rm{GF}} \right) = \frac{{W_{\rm{f}}}}{{W_{\rm{i}}}}$$

The crosslinking efficiency (CE) was quantitatively evaluated by high resolution magic-angle spinning (HR-MAS) ^1^H-NMR spectroscopy (Bruker Avance II 700 spectrometer, 700.13 MHz). The spectrometer included an HR-MAS probe which was equipped with an ^1^H, ^13^C, ^119^Sn and gradient channel. The spinning rate was set at 6 kHz. Constructs were placed in a 4 mm zirconium oxide MAS rotor (80 μL) after swelling in deuterated chloroform (40 µL). The crosslinking efficiency was calculated using Equation (6).

6 $${\rm{Crosslinking}}\,{\rm{efficiency}}\,\left( {\rm{CE}} \right)\,\left[ {{{\mathrm{\% }}}} \right] = \frac{{\left( {\frac{{I_{\rm{i}}}}{{I_{\rm{ri}}}} - \frac{{I_{\rm{e}}}}{{I_{\rm{re}}}}} \right)}}{{\left( {\frac{{I_{\rm{i}}}}{{I_{\rm{ri}}}}} \right)}}\times\,100{{{\mathrm{\% }}}}$$

with: *I*_i_ = integration of the acrylates before crosslinking (5.83–6.4 ppm). *I*_ri_ = integration of the reference peak (EO) before crosslinking (3.55–3.65 ppm). *I*_e_ = integration of the acrylates after crosslinking (5.83–6.4 ppm). *I*_re_ = integration of the reference peak (EO) after crosslinking (3.55–3.65 ppm).

##### Characterization of the viscoelastic properties using rheology

Viscoelastic characteristics of the AUP530 material in solution in different concentrations (i.e. 30 and 100 wt%) were measured with rheology. More precisely, the viscosity of the AUP material as well as the storage modulus (G′) were determined. For these tests, a volume of 280 µL was placed on the support plate (Anton Paar Physica MCR 301 rheometer with Rheoplus software). A PP25 spindle (i.e. D: 25 mm) was positioned above the electrospinning solution with a gap setting of 0.3 mm. All measurements were performed over a time of 20 min at 22 °C. After 500 s, samples were irradiated through a quartz glass bottom plate using UV-A light at 365 nm and an intensity of 7500 mW cm^−2^ (EXFO Novacure 2000 UV-light source). The measurements were carried out in triplicate (*n* = 3) with an exponentially increasing shear rate from 0.01 s^−1^ to 100 s^−1^. Viscosity (Pa s) was analyzed as a function of shear rate and the storage modulus G’ (Pa) was examined as a function of time (s).

### Material processing using electrospinning

#### Parameter optimization

##### Solution parameters

Both ES solutions, i.e. PCL (reference) and AUP530 (i.e. AUP530:PCL blend with a 50:50 ratio), were constituting a polymer concentration of 16 wt% in chloroform. The photo-initiator, ethyl (2,4,6-trimethylbenzoyl) phenyl phosphinate (TPO-L), was applied in a quantity of 2 mol% relative to the acrylate content to facilitate crosslinking in the post-electrospinning step. The ES solutions were analyzed via rheological measurements to determine the viscosity and storage modulus G’ (upon in situ crosslinking). The protocol for rheology has been described above (Section 2.1.2.4).

To enhance tendon repair, active compounds were incorporated in the ES constructs. These additional components were included in the polymer solution (PCL or AUP530:PCL) prior to ES. Based on literature [26, 28–31], the addition of an anti-adhesion and an anti-inflammatory component (i.e. HA, 1 wt% and Nap, 1.5 wt%) was pursued.

##### Device processing parameters

The in-house manufactured electrospinning set-up is composed of a high voltage source (Glassman High Voltage, Inc.; model series EL50P00, high voltage DC power), a motion controller (CWFW Ghent University) and a motor-driven syringe pumping system (New Era Pump Systems, Inc.; model Single Syringe Pump NE-300). The applied processing parameters (i.e. voltage: 15–20 kV, flow rate: 0.5–8.5 mL h^−1^ and needle-to-collector: distance 18–21 cm) were varied within the ES process and an optimal set of parameters was selected subsequently: voltage of 18 kV, flow rate of 1.5 mL h^−1^ and needle-to-collector distance of 18 cm. ES was performed at 21 °C and the relative humidity was determined by a hygrometer which was present in the ES cabinet.

The homogeneous polymer solution (stirred overnight) was transferred into a 20 mL syringe that was clamped into the syringe pumping system. The ES needle (inner diameter: 0.58 mm) was placed at a variable height above the collector. First, a flat plate collector was used to generate sheets and the quality of the fibers was assessed. Secondly, a mandrel rotating around its axis (180 rpm, Inox stainless steel, 4–6 mm diameter) was applied during the process of ES to produce tubular constructs. For an easy release of the electrospun tubes from the mandrel, preheated mandrels were dipcoated in molten poly(ethylene glycol) 8000 g mol^−1^ (PEG8k) and, after performing ES, were submerged in ultrapure water to dissolve the water-soluble PEG-coating and allow an easy release of the developed tubular constructs.

#### Post-processing photo-crosslinking step

After ES, AUP530:PCL mats and tubular constructs were transferred into a UV-transparent plastic bag and subsequently flushed with argon to remove oxygen that could interfere with the crosslinking process. The UV-treatment (both above and below the construct) was performed by UV-A irradiation at 10 mW cm^−2^ (Philips TL 0W/08 P8 T5/BLB lamps in the holder of Bi-Sonic Technology Corp.; model 8B-230 HB; 250–450 nm range) for 30 min.

### Mechanical evaluation of the developed repair construct

#### Tensile testing of developed drug-loaded electrospun tubes without reinforcement

**Fig. 1:**
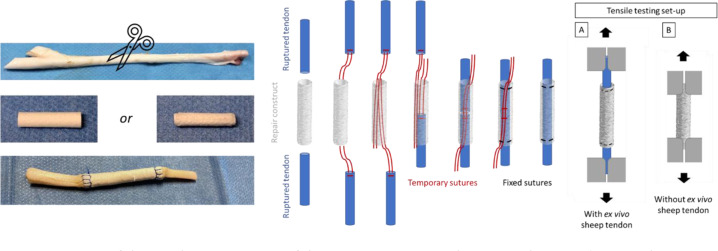
Set-up of the tensile testing set-up of the repair construct on the ex vivo sheep tendon. Tensile testing set-up: **A** with ex vivo sheep tendon, clamping the tendon ends in the tensile machine, **B** without ex vivo sheep tendon, clamping the repair constructs’ end in the tensile machine

The developed ES tubes (PCL as reference, compared to AUP530:PCL), with and without the incorporation of additional drugs, were tested for their mechanical strength by means of uniaxial tensile testing (Tinius Olsen 5ST, with Horizon software). The ES tubes (D: 5 mm) were compressed at their peripheral ends by the clamps (Fig. 1B) with a gauge length of 1.5 cm and were elongated in their longitudinal direction. The speed used for all configurations in the tensile tests was 10 mm min^−1^ with a preload tension of 0.1 N. The tensile tests were conducted in sixfold (*n* = 6).

#### Tensile testing of reinforced, drug-loaded electrospun tubes on ex vivo sheep tendons

First, an ex vivo tensile test on the non-reinforced, drug-loaded constructs was performed and the obtained data were compared to those of reinforced, drug-loaded constructs (PCL as a reference, compared to AUP530:PCL) applied in cadaveric sheep tendon. Indeed, this test is able to measure the strength by clamping the tendon ends, compared to the regular tensile test whereby the polymeric structure is clamped (as described above). An example of the applied set-up is shown in Fig. 1A.

Hind limbs of sheep were collected in a local abattoir. The flexor digitorum profundus tendons were immediately dissected from the sheep’s hind leg and frozen at −18 °C. Sheep tendons were chosen as a model because their tensile forces correspond mostly to those of human deep flexor tendons [32]. After thawing, a complete transection was induced in the middle of the tendons. Both ends of the transected tendon were pulled in the construct with a temporary suture at both ends (Fig. 1). Subsequently, either the non-reinforced constructs or the reinforced constructs were fixed at their outer ends to the tendon by a circumferential interlocking suture, again using a polydioxanone suture (PDS size 4-0, Ethicon Inc., Piscataway, NJ). The samples were mounted in a tensile testing machine (Lloyd Ametek C2S, software Nexygen Plus) with a clamp distance of 5 cm. After a preload of 1 N, an elongation test at a speed of 20 mm·min^−1^ was performed until failure. Each condition was tested in triplicate (*n* = 3).

#### Degradation study in aqueous environment

Tubular constructs (thickness = 1 mm, inner diameter = 5 mm and length = 0.5 cm) were electrospun, the initial mass of each sample was registered, and the samples were subsequently incubated (*T* = 37 °C, phosphate-buffered saline (PBS), pH = 7.4). Three samples were each time (every 2 weeks) removed and washed with ultrapure water to eliminate possible PBS salts. Samples were weighed and compared to their original mass. This ratio describes the percentage of mass loss and can thus be used as an indication of degradation. The degradation was studied for a time period of 17 weeks. The degradation study of the samples (*n* = 3) was performed every 2 weeks.

### Biological evaluation of the developed repair construct

#### Cytotoxicity assay using human fibroblasts

In vitro (in)direct cell tests, using commercial human fibroblasts (hFBs) (purchased from ATCC), were executed during this study to determine the biocompatibility of the materials. The electrospun materials (PCL and AUP530:PCL), as described in Table 1, were first evaluated without additional compounds (reference) and subsequently with the addition of Nap or HA. The effect of Nap and HA concentrations on cellular response and cell viability was evaluated. Samples were sterilized by incubation in 70% (v/v%) ethanol for 24 h (with a refreshing of ethanol after 12 h) and subsequent UV-C irradiation (100–250 nm, 15 mW cm^−2^) for 2 h.

**Table 1:** Overview of electrospun materials (PCL and AUP530:PCL) with or without additional components during in vitro (in)direct cell tests

Function	Component	PCL*	AUP530:PCL**
Reference	/	PCL Ref	AUP Ref
Anti-inflammatory	Nap	PCL Nap	AUP Nap
Anti-adhesion	HA	PCL HA	AUP HA
Positive control			

* = PCL (16 wt%), ** = AUP530 (8 wt%) PCL (8 wt%)

Human fibroblasts were cultured in culture medium, consisting of Dulbecco’s Modified Eagle Medium (DMEM) high glucose, 10 v% nutritional fetal bovine serum and 1 v% antibacterial penicillin/streptomycin (P/S), at 37 °C in 5% CO_2_ until a desired number of cells was reached. Culture medium was refreshed every 2–3 days, and cells were passaged when 80–90% confluency was reached. Passage 13 hFBs were used in these experiments. As positive control group, hFBs not having contact with the electrospun materials were included.

##### Indirect cell test

The developed repair constructs were incubated during 1, 3 and 7 days to assess the effect of potential leachables using a cell proliferation and viability assay. These experiments were performed in triplicate (*n* = 3). To this end, 10 000 hFBs were seeded onto a 96-well plate until 80% confluence.

The metabolic activity of the hFBs was assessed through an 3-(4,5-dimethylthiazol-2-yl)-5-(3-carboxy-methoxyphenyl)-2-(4-sulfophenyl)-^2^H-tetrazolium (MTS) assay. A mixture of 16 v/v% of MTS in culture medium was prepared and added to the hFBs. Next, the MTS was bioreduced into the colored formazan by incubation at 37 °C for 2 h in the dark (wrapped with aluminum foil) under continuous shaking. Thereafter, a spectrophotometer (BioTek Instruments; EL800 Universal Micropate Reader, with GEN5 software) was used to measure the absorbance of formazan at 490 nm.

Furthermore, a live-dead viability assay was carried out using calcein-acetoxymethyl/propidium iodide (Ca-AM/PI). A mixture of 0.2 v/v% Ca-AM and 0.2 v/v% PI in PBS was added to the hFBs and incubated in the dark by covering them with aluminum foil for 15 min at room temperature. Visualization of the HUVECs was performed by fluorescence microscopy (Olympus IX 81 with software Xcellence Pro), using a fluorescent protein (GFP) filter and a Texas Red (TxRed) filter, which led to the distinction of green living cells and red dead cells.

##### Direct cell test

A biocompatibility assay was conducted on cells which were in direct contact with the electrospun material. After sterilization, the materials were added to a confluent monolayer of hFBs in a 48-well plate (30,000 cells/well) supplemented with 400 μL culture medium. Cell viability was assessed in triplicate (*n* = 3) by a live-dead Ca-AM/PI fluorescence staining as well as a cell proliferation assay using MTS after 1, 3 and 7 days.

#### Indirect co-culture (tenocytes and MSCs) assay to evaluate collagen production

To quantify the total collagen production, a Sirius Red/Fast Green Collagen staining kit (Chondrex) was used in mono-cultures (equine tenocytes or MSCs, respectively) and compared to direct co-cultures (equine tenocytes and MSCs). This was tested on electrospun materials (PCL and AUP530:PCL) without additional components (reference) as well as supplemented with Nap or HA (Table 1).

Both tenocytes and MSCs were harvested from a 15-year-old Arabian horse. Equine tenocytes were isolated from the superficial digital flexor tendon using 0.1% collagenase type Ia digestion in high glucose DMEM [33]. MSCs were collected from abdominal adipose tissue using 0.1% liberase digestion in low glucose DMEM. Both cell types were cultured until passage 4 and were then seeded in 12-transwell plates at a density of 25,000 cells cm^−2^ in tenocyte medium (consisting of HG-DMEM, 10% FBS, 1% l-glutamine, 1% P/S/Amphotericin B(A)) or MSC culture medium (consisting of LG-DMEM, 30% FBS, 1% l-glutamine, 1% P/S/A), respectively. In addition, a co-culture with a ratio of 50% tenocytes and 50% MSCs was included in this study, cultured in a 50:50 ratio of the corresponding media, as well as a tissue culture plastic (TCP) control.

ES discs (D: 8 mm) were punched out and sterilized before cell seeding. ES discs were placed on a transparent insert with a pore size of 0.4 µm. Cells (i.e. tenocytes and/or MSCs) were located at the bottom of the well in order to have an indirect contact between the disc and the cells (see Fig. S2). All experiments were performed in triplicate (*n* = 3).

The Sirius Red staining kit was used to quantify collagen production. In brief, medium was discarded after 7 days, and cells were washed with PBS. An amount of 0.5 mL of Kahle fixative (i.e. 60 v% distilled water, 28 v% 97% ethanol, 10 v% 37% formaldehyde and 2 v% glacial acetic acid) was added and subsequently incubated for 10 min at room temperature. After removing the fixative and washing the cells again with PBS, 250 µL of dye solution was added and incubated at room temperature for 30 min. The stained cell layers were rinsed three times with distilled water followed by dehydration in ethanol from 70% to 96% (1 min per step). After visualization, 1 mL of dye extraction buffer was loaded in each well and carefully mixed by pipetting until the color was eluted from the sample. Finally, 100 µL was compiled of the eluted dye solution and transferred into a 96-well plate. The optical density (OD) was measured by a spectrophotometer (Infinite F50 Tecan) at 550 nm and 595 nm. The total collagen and non-collagenous protein production were determined by Equations (7) and (8).

7 $${\rm{Total}}\,{\rm{collagen}}\left[ {\frac{{{{{\mathrm{\mu }}}}\rm{g}}}{{\rm{section}}}} \right] = \frac{{{\rm{OD550}}\,{\rm{value}} - \left( {{\rm{OD595}}\,{\rm{value}}\,\times\,0.291} \right)}}{{{\rm{Col.}}\,\rm{Eq}}}$$

8 $${\rm{Non}}-{\rm{collagenous}}\,{\rm{proteins}}\,\left[ {\frac{{{{{\mathrm{\mu }}}}{\rm{g}}}}{{\rm{section}}}} \right] = \frac{{\rm{OD595}}}{{{\rm{Col.}}\,{\rm{Eq}}}}$$

with: OD550 value = optical density of Fast Green (550 nm). Contribution of Fast Green at 550 nm is 29.1% → OD595 value is multiplied by 0.291. OD595 value = optical density of Sirius Red (595 nm). Col. Eq (total collagen) = color equivalence of total collagen = 0.0378. Col. Eq (non-collagenous proteins) = color equivalence of non-collagenous proteins = 0.00204.

### Statistical analysis

The mean and standard deviation (SD) on the samples (at least triplicate) were calculated using GraphPad Prism 7 software and all data were expressed as mean ± SD. Statistical evaluation was carried out by performing a one-way or two-way ANOVA, with a post Tukey multiple comparison test. Differences were considered statistically significant at *p* < 0.05 and were annotated with *.

## Results

### Material synthesis and characterization

**Fig. 2:**
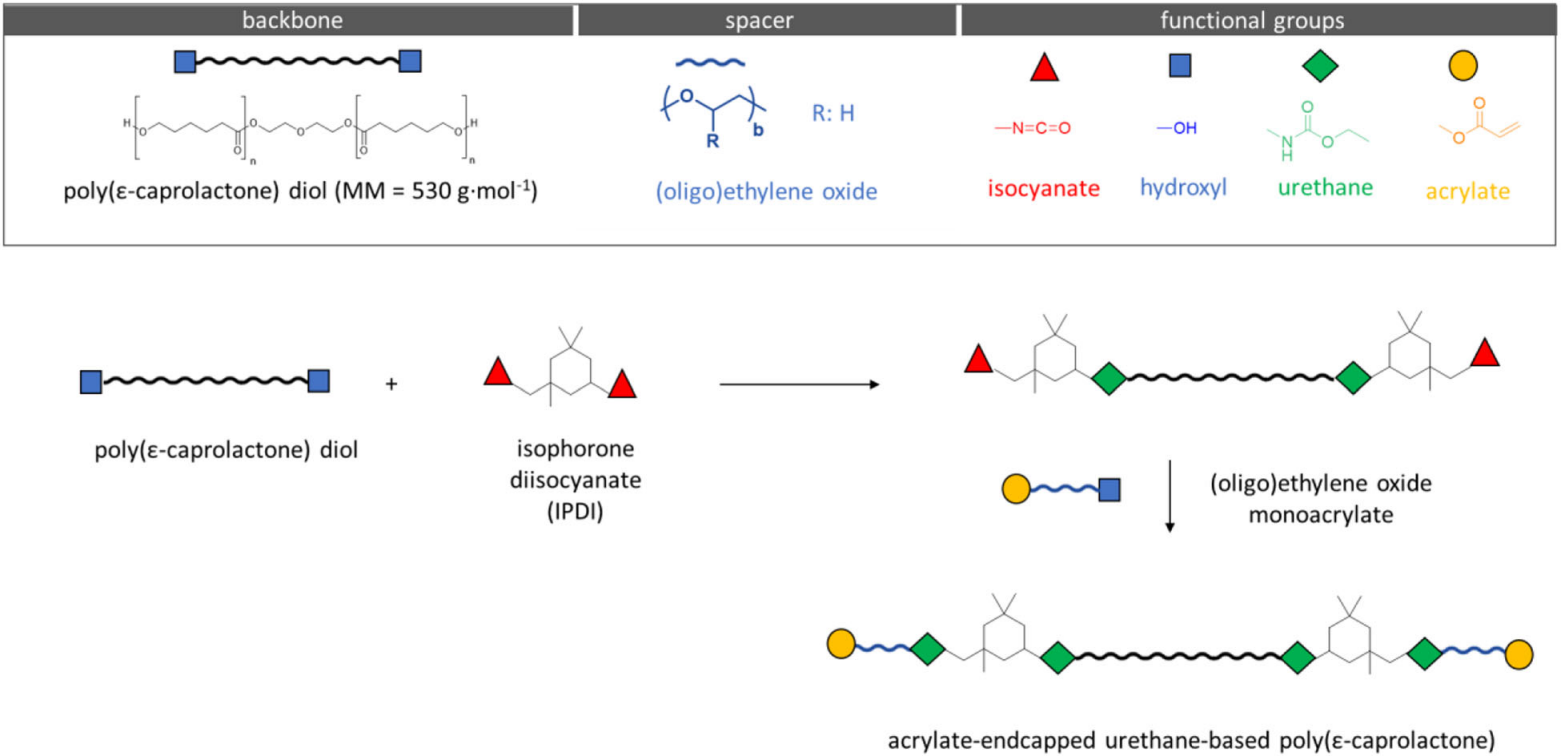
Acrylate-endcapped urethane-based polymer (AUP) synthesis overview

A novel acrylate-endcapped urethane-based material based on a PCL backbone has been synthesized to develop a tubular construct for tendon repair. AUP precursors were created by attaching at both ends of the central backbone polymer, a diisocyanate linker connected to a short flexible oligomer spacer (ethylene oxide) [34, 35] that separates the backbone from the photoreactive acrylate end-groups. Considering the requirements with regard to mechanical strength for the intended application of tendon repair, a hydrophobic PCL backbone with a MM of 530 g mol^−1^ was selected (Fig. 2).

^1^H-NMR spectroscopy (Fig. S1) enabled the calculation of the the acrylate content and the MM, and indicated a MM for the PCL diol (585 g mol^−1^), a MM for the AUP (1652 g mol^−1^), and an acrylate content of 0.983 mmol g^−1^. The latter enabled the calculation of the amount of photo-initiator required to be included in the electrospinning solutions (i.e. 2 mol% with respect to the acrylate content).

The thermal properties of the AUP530 precursor were evaluated using TGA and DSC analysis (Fig. S3). The TGA analysis indicated that a 1% mass loss occurred at 153 °C, whereas 5% of the polymer was degraded at 263 °C, and complete degradation occurred at 406 °C. No crystallization nor a melt peak were detected in the DSC thermograms, whereas a glass transition peak could be observed at −40 °C.

**Fig. 3:**
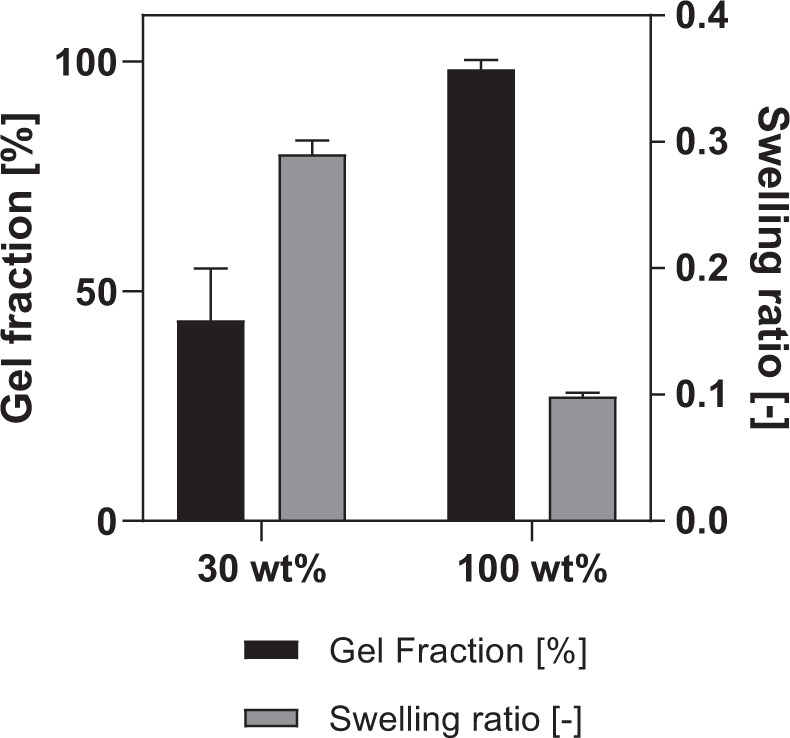
Measured swelling ratio and gel fraction of AUP530 at two different concentrations (i.e. 30 and 100 wt%)

Next, the crosslinked AUP material was physico-chemically characterized by determining the swelling ratio, gel fraction and crosslinking efficiency. A negligible swelling ratio for both AUP530 crosslinked materials (i.e. 0.3 ± 0.01 and 0.1 ± 0.02, for the 30 and 100%, respectively) (Fig. 3) was obtained. In addition, a gel fraction of almost 100% (i.e. 98.3 ± 2.0%) was obtained for the AUP530 (100 wt%). The AUP530 (30 wt%) exhibited a significantly lower (*p* < 0.05) gel fraction (i.e. 43.7 ± 0.2%). A crosslinking efficiency of 92.9% was obtained by HR-MAS NMR spectroscopy for the AUP530 100 wt%.

The viscoelastic properties were determined using rheology. Rheological measurements were executed in the absence of a photo-initiator in order to evaluate the crosslinking ability of the developed material itself (whereas a photo-initiator will be added to the electrospinning solution to facilitate photo-crosslinking after processing). The storage modulus was plotted on a logarithmic scale as a function of time for various AUP530 concentrations (i.e. 30 and 100 wt%) (Fig. S4, Table S1). The samples demonstrated a high increase in storage modulus upon UV-curing. AUP530 (30 and 100 wt%) showed a storage modulus of 501 ± 11 and 1.773 ± 0.029 MPa, respectively. The viscosities of the AUP530 concentrations at a shear rate of 60 s^−1^ are shown in Table 2. AUP530 (100 wt%) showed a significantly higher viscosity (i.e. 91.70 ± 2.72 Pa s) (*p* < 0.05) compared to the 30 wt% solution (i.e. 0.17 ± 0.11 Pa s).

**Table 2:** Overview of the measured storage moduli and viscosity for various AUP concentrations as obtained using rheology and in situ UV crosslinking

AUP concentration	Storage modulus [kPa]	Viscosity [Pa s]
30 wt%	501 ± 11	0.17 ± 0.11
100 wt%	1 773 ± 29	91.70 ± 2.72

### Design of the repair construct and development of the tubular constructs using electrospinning

**Fig. 4:**
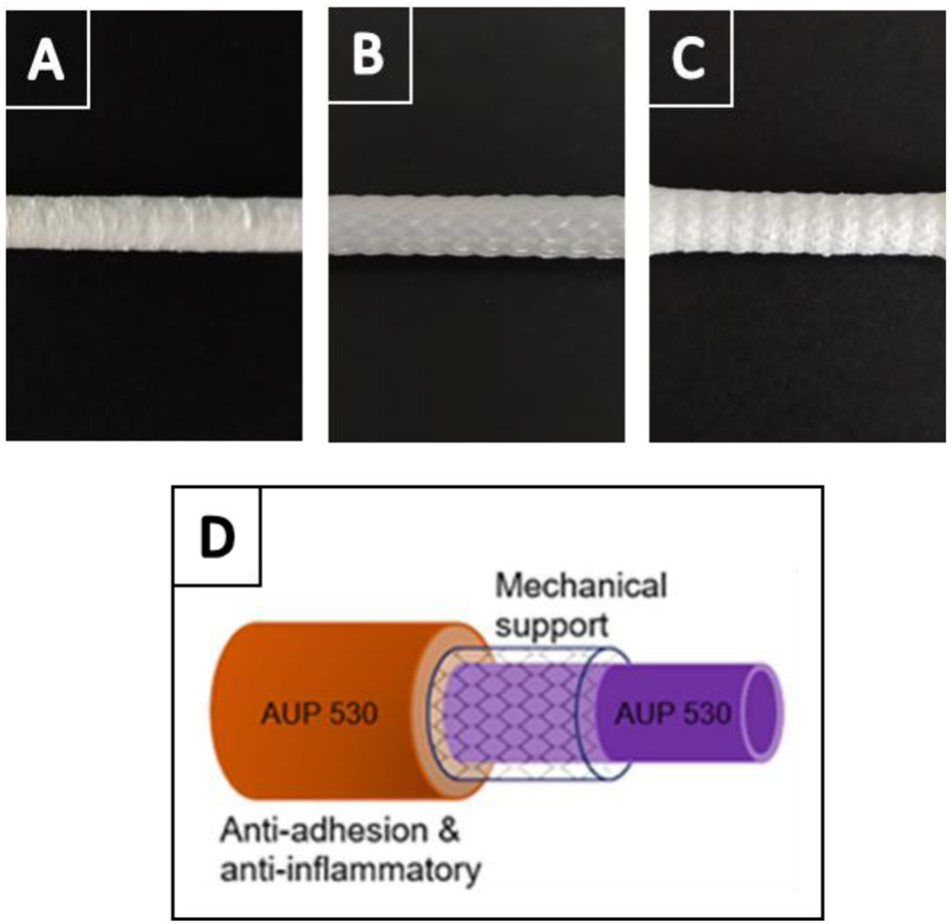
Visualization of a reinforced, drug-loaded ES repair construct. **A** Inner layer with no additional drugs that serves at enclosing the tubular braid in between two electrospun layers. **B** Tubular braid with a Chinese finger trap mechanism that acts as a mechanical support. **C** Outer layer with incorporated anti-adhesion and anti-inflammatory components. **D** Schematic visualization of the multi-layered repair construct. PCL was used as a reference

The design of the tubular repair constructs in the present study is based on multiple layers. The inner layer and outer layer of the construct were electrospun AUP530:PCL or PCL (as a reference) layers. The outer layer included anti-adhesion and anti-inflammatory drugs, i.e. hyaluronic acid (HA) and Naproxen (Nap). The middle layer acts as a reinforcement layer in between the two ES layers. This reinforcement is based on the Chinese finger trap mechanism and is composed of a polypropylene tubular braid. The proposed reinforced, drug-loaded electrospun construct design is depicted in Fig. 4.

### Mechanical evaluation of the developed repair construct

#### Tensile testing of developed drug-loaded electrospun tubes without reinforcement

Tensile testing indicated a Young’s modulus of 29.6 ± 11.6 MPa and an ultimate stress of 10.6 ± 4.3 MPa for the drug-loaded AUP530:PCL repair constructs. No significant differences were observed when comparing the mechanical properties of the drug-loaded versus the non-drug-loaded constructs (Table 3). However, a significant increase (*p* < 0.05) was found in the ultimate stress value of the drug-loaded AUP530:PCL compared to the non-drug and the drug-loaded PCL repair construct.

**Table 3:** Overview of the obtained Young moduli (MPa) and ultimate stresses (MPa) for ES constructs

Non-drug loaded vs drug-loaded	Material	Young’s modulus [MPa]	Ultimate stress [MPa]
Non-drug loaded ES construct	PCL	22.2 ± 4.3	4.2 ± 1.2
Drug-loaded ES construct	PCL	18.9 ± 5.9	3.3 ± 0.5
Non-drug loaded ES construct	AUP530:PCL	15.4 ± 3.1	6.2 ± 1.4
Drug-loaded ES construct	AUP530:PCL	29.6 ± 11.7	10.6 ± 4.3

#### Tensile testing of reinforced, drug-loaded electrospun tubes on ex vivo sheep tendons

**Fig. 5:**
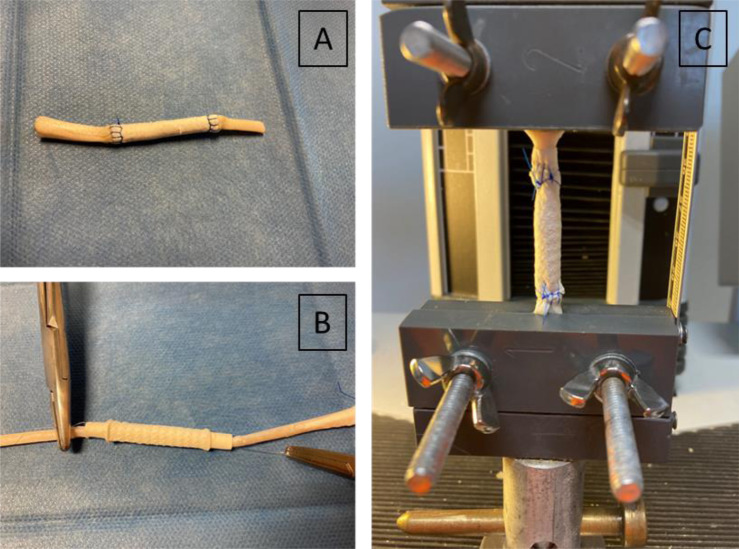
Ex vivo tensile testing of the repair constructs using cadaveric sheep tendons (**A**) repair construct without reinforcement; **B**, **C** reinforced repair construct

In addition to arbitrary tensile testing of the repair construct, also ex vivo tensile tests were performed on cadaveric sheep tendons. The tensile testing set-up is shown in Fig. 5. Both non-reinforced and reinforced repair constructs were tested (Fig. 5). The non-reinforced repair constructs failed at an average ultimate stress of 0.4 ± 0.1 MPa and 0.8 ± 0.4 MPa for the PCL and AUP530:PCL, respectively. Whereas the developed reinforced repair constructs attained ultimate stresses of 4.8 ± 1.0 MPa and 6.4 ± 0.6 MPa for PCL and AUP530:PCL. The reinforced AUP530:PCL repair construct showed a statistically significant higher maximum load (126.2 ± 10.9 N) compared to the reinforced PCL repair construct (95.1 ± 18.7 N). In between the materials, there was a significant difference (*p* < 0.05) when comparing the non-reinforced to the reinforced repair constructs, both with regard to the Young’s moduli and the ultimate stresses (Table 4).

**Table 4:** Overview of the tensile testing data of the non-reinforced, drug-loaded versus reinforced, drug-loaded repair constructs (AUP compared to PCL as a reference) on ex vivo sheep tendons

Non-reinforced vs reinforced	Material	Young’s modulus [MPa]	Ultimate stress [MPa]
Non-reinforced drug-loaded	PCL	0.3 ± 0.2	0.4 ± 0.1
Reinforced drug-loaded	PCL	5.7 ± 1.2	4.8 ± 1.0
Non-reinforced drug-loaded	AUP530:PCL	2.5 ± 0.9	0.8 ± 0.4
Reinforced drug-loaded	AUP530:PCL	9.4 ± 2.5	6.4 ± 0.6

#### Degradation study in aqueous environment

**Fig. 6:**
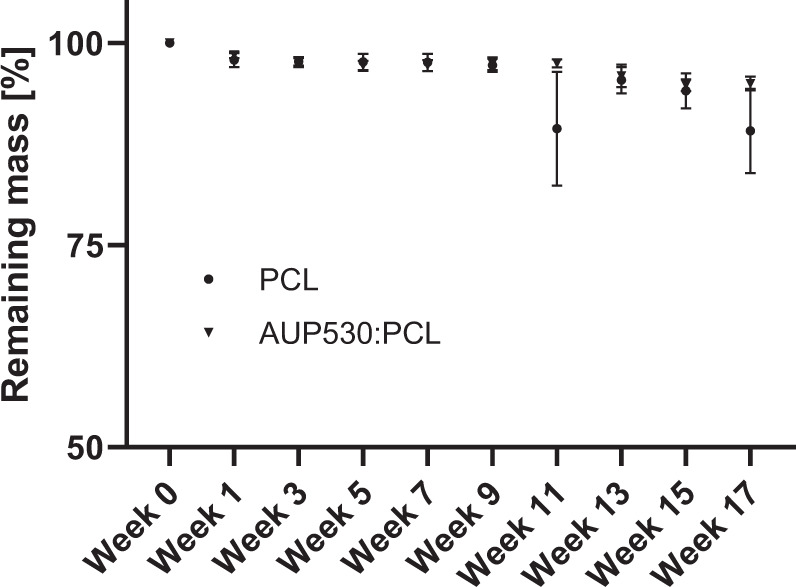
Degradation study of the electrospun AUP and PCL repair constructs in aqueous medium

A degradation study in phosphate-buffered saline (PBS) was conducted on the developed electrospun repair constructs (AUP530:PCL, and PCL as reference). Figure 6 demonstrates the degradation of the electrospun repair constructs, based on the remaining mass in function of the number of weeks. Only after 10 weeks, a decrease in remaining mass was observed, more in the PCL repair constructs than the AUP530:PCL repair constructs. The mass of the repair constructs did not significantly (*p* > 0.05) decrease up to 10 weeks.

### Biological evaluation of the developed repair construct

#### In vitro biocompatibility evaluation of the developed material

Potential cytotoxicity of the developed drug-loaded electrospun constructs was assessed using both an indirect and direct contact test. Evaluation of the biocompatibility of the materials was performed by Ca-AM/PI staining and an MTS assay at three different time points (i.e. day 1, day 3 and day 7) both in indirect and direct contact with human fibroblasts (hFBs).

**Fig. 7:**
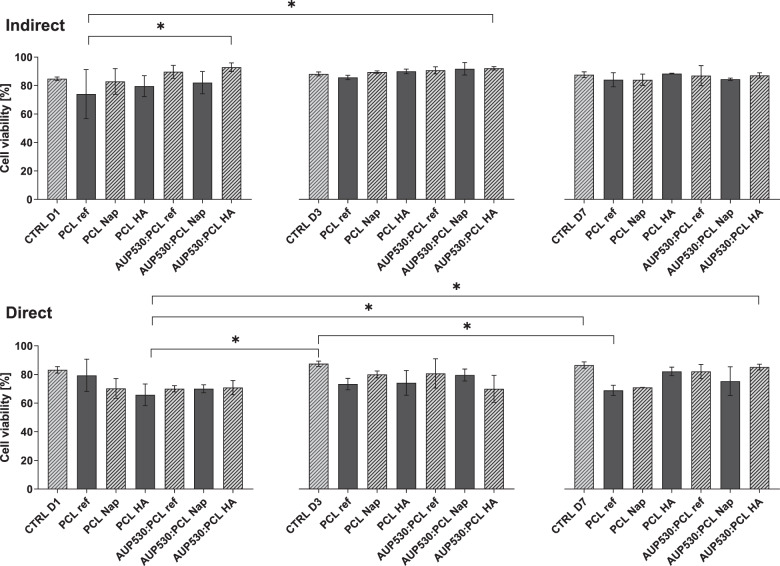
Cell viability of indirect (top) and direct (bottom) assay using hFBs, by a Ca-AM/PI staining at day 1, 3 and 7. (*p < 0.05). Tissue culture plastic was used as a positive control

Using the Ca-AM/PI staining, it was demonstrated that cell viability for all materials in both contact tests exceeded 70% after 7 days (Figs. 7 and S6). Moreover, viability data for these materials was non-significantly different when compared to the tissue culture plastic positive control. The same trend was observed for the metabolic activity, used as a marker of cell proliferation (Fig. S5).

#### Quantification of the total collagen production

**Fig. 8:**
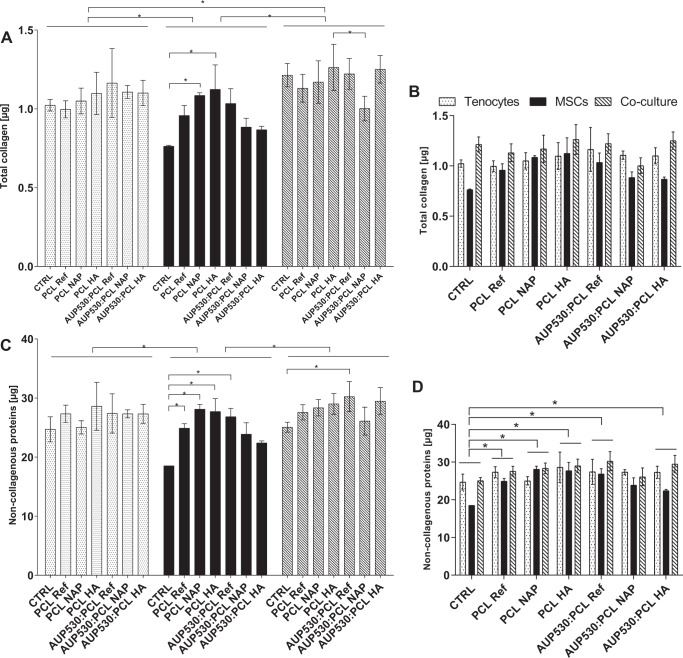
Production of total collagen (**A**, **B**) and non-collagenous proteins (**C**, **D**) illustrated in mono-cultures (tenocytes or mesenchymal stem cells, MSCs) and co-culture (tenocytes & MSCs) after an incubation of 7 days, including overall effects (**B**, **D**). Cells were cultured in direct contact with the electrospun constructs (PCL and AUP530:PCL). Tissue culture plastic was used as a positive control. (*p < 0.05)

The total collagen production was quantified using a mono- (tenocytes or MSCs) or a co-culture system. After 7 days, the main collagen production was significantly higher (*p* < 0.05) in the co-culture system (tenocytes & MSCs) when compared to mono-culture systems (tenocytes or MSCs) (Fig. 8). When considering only the mono-cultures, a significantly higher (*p* < 0.05) collagen production was observed in tenocytes when compared to MSCs (Fig. 8A). Additionally, a high amount of non-collagenous proteins was detected in all cell conditions when compared to the total collagen production, and this production was significantly higher (*p* < 0.05) in the co-culture system when compared to MSC mono-culture, indicating that both collagen and non-collagenous protein production are higher in the co-culture system (Fig. 8C). The Sirius Red/Fast Green staining images are depicted in Fig. S7.

In the MSC mono-culture system, collagen production was significantly increased when MSCs were cultured on PCL Nap and PCL HA (*p* < 0.05) when compared to TCP. In the co-culture system, collagen production was significantly higher in PCL HA when compared to AUP530:PCL Nap (*p* < 0.05).

Regardless of the cell type, the collagen production, did not significantly differ between the materials evaluated in this study (Fig. 8B). In almost all materials, except for AUP530:PCL Nap, however, an overall significantly higher (*p* < 0.05) production of non-collagenous proteins was observed when compared to TCP (Fig. 8D). These findings were confirmed in the MSC mono-culture, except for AUP530:PCL HA (Fig. 8C). In the co-culture, only AUP530:PCL ref showed a significantly higher non-collagenous protein production when compared to TCP (*p* < 0.05).

## Discussion

In this study, a novel acrylate-endcapped urethane-based material was synthesized. One of the main advantages of AUPs is that they enable crosslinking in the solid state. This is especially interesting when the material is processed by electrospinning, and (near) solvent-free fibers are obtained after the ES process. By means of a post-processing step entailing UV irradiation, the AUP fibers can thus be crosslinked, resulting in superior mechanical strengths for the developed tubular repair constructs. The decision to start from a MM 530 g mol^−1^ PCL backbone (AUP530) was mainly to attain the specific mechanical properties required for tendon repair (*vide infra*).

Because the repair construct will be introduced into the human body, it is critical to evaluate at which temperature range the newly developed polymer remains stable. The TGA analysis indicated that complete degradation occurred at 406 °C. This is in accordance with other PCL-based materials as they appear to be stable up to 380 °C and completely degrade around 410 °C [36]. Based on its thermal properties, the developed AUP530 precursor is suitable for the proposed application, i.e. tendon repair (and electrospinning processing), but also for processing by other techniques including 3D printing, because the materials can be used without encountering a degradation risk upon exposure to the elevated processing temperatures.

In a next step, the physico-chemical properties of the developed material were determined. On the one hand, the swelling ratio was determined to evaluate the material’s ability to absorb water (as a simplification of the body’s physiological fluids). In case of application in tendon repair, swelling of the construct should be minimized because this can compress the surrounding tissues and thus impair the effect of healing. As expected, the hydrophobic behavior of the AUP530 polymer (due to the PCL backbone), led to a negligible swelling ratio for both AUP530 crosslinked materials (i.e. 0.3 ± 0.01 and 0.1 ± 0.02, for the 30 and 100% materials, respectively) (Fig. 3). On the other hand, the gel fraction gives a first indication of the crosslinking efficiency, and is ideally very high (close to 100%) in order to avoid the release of non-crosslinked compounds inside the body. The crosslinking efficiency was further evaluated and more precisely confirmed by HR-MAS ^1^H-NMR spectroscopy. A gel fraction of almost 100% (i.e. 98.3 ± 2.0%) was obtained for AUP530 (100 wt%), indicating an excellent crosslinking efficiency. AUP530 (30 wt%) exhibited a significantly lower (*p* < 0.05) gel fraction (i.e. 43.7 ± 0.2%), showing that an AUP concentration of 30 wt% was not sufficient to obtain efficient crosslinking. The high values for the gel fraction for AUP 100 wt% are in agreement with the crosslinking efficiency (i.e. 92.9%) obtained by HR-MAS NMR spectroscopy for AUP530 100 wt%. This implies that almost all acrylates have reacted by the crosslinking mechanism and formed a crosslinked network, which is an important aspect to consider when the material will be used in tissue engineering applications.

The viscoelastic properties of a material are important toward material processing, especially in the electrospinning process. The samples demonstrated a high increase in storage modulus upon UV-curing, confirming the consumption of the acrylate functionalities endcapping the AUP530 polymer, during the photo-crosslinking process. AUP530 (30 and 100 wt%) showed a storage modulus of 501 ± 11 and 1.773 ± 0.029 MPa, respectively. As expected, compared to reported data on the storage modulus of a PCL-based AUP with a higher MM of 2000 g mol^−1^ (i.e. G′ = 0.7 MPa), AUP530 showed a higher storage modulus [37]. As shown in Table 2, AUP530 (100 wt%) showed a significantly higher viscosity (i.e. 91.70 ± 2.72 Pa s) (*p* < 0.05) compared to the 30 wt% solution (i.e. 0.17 ± 0.11 Pa·s). The polymer viscosity and/or concentration has a direct effect on the fiber size and morphology [38]. Therefore, this parameter was further evaluated and optimized with respect to the processing potential of the AUP material and the optimized processing parameter set (Sections 2.2 and 3.2).

After characterization of the developed AUP530 precursor, the processing potential was evaluated using solution electrospinning, and the processing and material parameters were optimized to obtain homogeneous electrospun fibers without beads. The design of the tubular repair constructs in the present study is based on multiple layers, and depicted in Fig. 4. The inner layer and outer layer of the construct were electrospun AUP530:PCL or PCL (as a reference) layers. The outer electrospun layer included anti-adhesion and anti-inflammatory drugs, i.e. hyaluronic acid (HA) and Naproxen (Nap). These drugs should counter post-surgical peritendinous inflammatory responses and adhesion formation with surrounding tissues, which are known to induce pain and impede a proper healing and poor (re-)functionality of the tendon [27]. The middle layer, based on the Chinese finger trap mechanism, acts as a reinforcement layer in between the two ES layers. This type of tubular braid tightens and grasps the tendon upon elongation, mimicking the stress-strain behavior of tendons [15].

One of the main goals was to develop a repair construct with sufficient mechanical properties to support the tendon in the first stages of its recovery process to improve healing. To evaluate the characteristics of the developed electrospun repair construct, mechanical testing of the repair constructs (drug-loaded versus non-drug loaded) was performed. Additionally, the mechanical properties of the repair construct were evaluated using ex vivo sheep tendons. The developed AUP530:PCL repair constructs were compared to PCL repair constructs (as a reference). The minimal stress the construct should be able to bear in order to obtain an optimal repair, is approximately 4 MPa in hand tendons [17].

In this research, the decision to start from a MM 530 g·mol^−1^ PCL backbone (AUP530) was mainly to attain superior mechanical properties when compared to AUPs with higher MM PCL backbones. When comparing the AUP530 with an AUP with a higher MM PCL backbone of 2000 g·mol^−1^ (AUP 2000), an increase in Young’s modulus (29.6 ± 11.6 MPa) compared to AUP 2000 (6.7 ± 2.3 MPa) was observed [39]. This was expected as shorter polymer chains lead to a more densely crosslinked network, increasing the stiffness. Moreover, the ultimate stresses measured for the drug-loaded AUP530:PCL (without ex vivo tendon and without suturing) (i.e. 10.6 ± 4.3 MPa) exceeded the threshold of 4 MPa for flexor tendon repair [17]. A significant increase (p < 0.05) was found in the ultimate stress value of the drug-loaded AUP530:PCL compared to the non-drug and the drug-loaded PCL repair construct, implying superior mechanical properties of the drug-loaded AUP530:PCL repair constructs compared to the PCL constructs (Table 3).

In addition to arbitrary tensile testing of the repair construct, ex vivo tensile tests were performed on cadaveric sheep tendons. Both non-reinforced and reinforced repair constructs were tested (Fig. 5). The non-reinforced repair constructs failed at an average ultimate stress of 0.4 ± 0.1 MPa and 0.8 ± 0.4 MPa for the PCL and AUP530:PCL, respectively. These ultimate stress values are not sufficient for the intended tendon repair application, as a minimum of 4 MPa should be targeted [17]. However, the developed reinforced repair constructs attained ultimate stresses of 4.8 ± 1.0 MPa and 6.4 ± 0.6 MPa for PCL and AUP530:PCL. The reinforced AUP530:PCL repair construct showed a statistically significant higher maximum load (126.2 ± 10.9 N) compared to the reinforced PCL repair construct (95.1 ± 18.7 N). Moreover, the electrospun AUP530:PCL showed easier handling when hydrated, compared to the PCL repair constructs. There was a significant difference (p < 0.05) when comparing the non-reinforced to the reinforced repair constructs, both with regard to the Young’s moduli and the ultimate stresses (Table 4). The differences in Young’s moduli and ultimate stresses in the repair constructs, as described in the previous paragraph (Table 3) without ex vivo sheep tendons, and those in the mechanical testing using ex vivo tendons (Table 4), are due to the sutures that are used to attach the tendon ends to the repair construct in the latter. These sutures lead to more specific loads and stresses in the suture points instead of a more homogeneously spread load on the full repair construct as is the case when the repair construct is tested as described above (Section 3.3.1).

Haussman et al. [32]. studied two different surgical suturing techniques on human flexor tendons and on sheep, pig and calf tendons. They reported an ultimate stress of 2.05 ± 0.32 MPa for a Modified Kessler suture (currently the gold standard in tendon repair) and 3.47 ± 0.58 MPa for a deep running suture, exploited to repair sheep tendons (with a surface value of 19 mm^2^) [32]. Two other suturing techniques (i.e. Adelaide and Modified Adelaide) were evaluated by Tahmassebi et al. [40], and led to ultimate stresses of 2.56 ± 0.31 MPa and 2.73 ± 0.16 MPa respectively. Considering the average maximum failure stresses in the current study of 4.8 ± 1.0 MPa and 6.4 ± 0.6 MPa for PCL and AUP530:PCL respectively, the repair constructs reach superior ultimate stresses compared to most standard surgical suturing techniques. This suggests that the proposed repair technique might allow faster active rehabilitation, which is also beneficial in the prevention of adhesion formation.

The body-implanted construct should encourage a healing process (ideally within 6–8 weeks) in order to resume and stimulate the normal properties of a tendon [41]. Also, the tendon itself should be mechanically strong enough to perform its function before the repair construct starts degrading and the healed tendon is strong enough to bear loads again. This implies that the repair construct should not start to degrade before a period of at least 8 weeks. Moreover, it has been reported that the degradation of PCL occurs through hydrolysis of its ester linkages upon contact with physiological conditions (such as the human body) [42]. This is associated with a reduction in strength and a decrease in mass as a result of fiber rupture. Therefore, a degradation study in phosphate-buffered saline (PBS) of more than 8 weeks was proposed to investigate whether premature mass loss of the electrospun repair construct (AUP530:PCL, and PCL as reference) would occur. The high standard variations obtained in this degradation study may be due to the limitation of accurate weighing of the low mass electrospun tubes (i.e. mg range). Only after 10 weeks, a decrease in remaining mass was observed, which was higher for the PCL repair constructs compared to the AUP530:PCL repair constructs. Importantly, the mass of the repair constructs did not significantly (*p* > 0.05) decrease in the crucial period up to 9 weeks, and thus would give the tendon enough time to pass to an advanced healing stage.

In tendon repair, adhesion is one of the main complications of the currently used surgical techniques and refers to the abnormal adherence of soft tissue to the surrounding structures [43]. In addition, during the repair period, large numbers of inflammatory cells are attracted to the injury site [27]. As a result, recurrent injury or loss of function are often observed. Therefore, the main goal of this research was to develop a mechanically and biologically relevant tubular construct for tendon repair. Within the biological approach, the main aim was to incorporate drugs, i.e. Nap and HA, in the electrospun construct to counter these adhesion and inflammatory processes. It has been reported in literature that Nap prevents adhesions by inhibiting the inflammatory response [28]. HA on the other hand is a natural polymer, ubiquitously present in the human body which has the ability to interfere with pro-inflammatory factors such as prostaglandins and cytokines [26]. Therefore, both drugs indirectly influence each other while no adverse effects on biocompatibility are expected [27].

It was demonstrated that cell viability for all materials in both indirect and direct contact tests exceeded 70% after 7 days (Figs. 7 and S6). Moreover, viability data for these materials were non-significantly different when compared to the tissue culture plastic control. Hence, the developed AUP530:PCL material, and upon incorporation of both an anti-inflammatory and an anti-adhesion component (i.e. Nap and HA) showed excellent biocompatibility, both in terms of the material on its own (direct contact) and regarding leachables (indirect contact) [44]. This was anticipated since previous research on PCL and AUP materials (with a higher MM) has already evidenced biocompatibility [15, 39].

In vivo, tenocytes, which are the terminally differentiated cells of a tendon, maintain extracellular matrix homeostasis and increase the production of collagen type I during the tendon healing process [45, 46]. MSCs represent a promising regenerative treatment for tendon injuries, as they are known to promote tissue regeneration, prevent pathological scar formation, modulate immune responses and regulate inflammation [47, 48]. As such, it has recently been demonstrated that direct co-culture of MSCs and tenocytes resulted in an increased proliferation and collagen type I production when compared with mono-culture controls [48]. Additionally, non-collagenous proteins such as glycoproteins, play an important role in modulating collagen fibrillogenesis during tendon development and healing [49]. Therefore, a co-culture with a ratio of 50% equine tenocytes and 50% equine MSCs was included in this study (and compared to the mono-culture systems of each cell type). In the MSC mono-culture system, collagen production was significantly increased when MSCs were cultured on PCL Nap and PCL HA (*p* < 0.05) when compared to TCP. As it is known that the micro-environment regulates stem cell function [50], the increased collagen production might be a result of the different biochemical micro-environment with the presence of Nap and HA, and a differing mechanical strength [51]. It should be mentioned however that the Sirius Red/Fast Green Collagen staining kit cannot distinguish between different types of collagen. In both materials (PCL and AUP530:PCL) evaluated in this study, an overall increased production of collagen and non-collagenous proteins was observed when tenocytes were directly co-cultured with MSCs. These findings illustrate that patients suffering from tendon injuries may benefit from MSC therapy as these proteins play a key role in tendon repair [48].

In this study, the addition of Nap and HA is suggested to improve the anti-adhesive and anti-inflammatory properties of the construct. However, this has not been confirmed experimentally. In a follow-up study, it would be interesting to investigate both the anti-adhesion and anti-inflammatory properties of the developed repair constructs in greater detail. In addition, a drug-release study would also provide more information on the release rate of the incorporated drugs. In future work, it would be interesting to evaluate other AUP chemistries to avoid the need for a reinforcement layer to ensure sufficient mechanical properties when sutures are being used. Alternatively, the development of a reinforcement layer based on a biodegradable material could be pursued. It should also be noted that in future experiments, the degradation study could be executed by combining monitoring the mass loss with mechanical testing. As a final point, although sheep tendons exhibit tensile forces that correspond mostly to those of human deep flexor tendons [32], the use of ex vivo human tendons instead of ex vivo sheep tendons could provide even more relevant information regarding potential translation toward the clinic.

## Conclusion

In the present study, both a mechanical and a biological approach was combined to develop a tubular electrospun repair construct for flexor tendon. In order to improve the healing process, an optimal and controlled healing environment should be provided to minimize inflammation and adhesion processes. To this end, an AUP with a PCL backbone of 530 g mol^−1^ was synthesized. ^1^H-NMR spectroscopy indicated a MM of the PCL diol of 611 g mol^−1^, a MM of the AUP of 1728 g mol^−1^, and an acrylate content of 0.973 mmol g^−1^. Physico-chemical characterization demonstrated that the AUP530 remained stable up to 263 °C (5% mass loss), had a high gel fraction (99.9 ± 1.0 %) and excellent crosslinking efficiency (92.9%). Subsequently, the AUP material was electrospun into a tubular repair construct. The repair constructs were mechanically tested using ex vivo sheep tendons, and have proven to fulfill the required mechanical properties for flexor tendon repair (i.e. minimal ultimate stress of 4 MPa). The reinforced AUP530:PCL repair construct showed a maximum load of 126.2 ± 10.9 N, a Young’s modulus of 9.4 ± 2.5 MPa and an ultimate stress of 6.4 ± 0.6 MPa. In addition, the repair constructs did not show any significant degradation before min. 8 weeks, which covers the initial healing period of an injured tendon to resume its normal properties and function. Moreover, anti-inflammatory and anti-adhesion components were incorporated to further optimize the repair construct, i.e. Nap and HA. In vitro biological evaluation using hFBs indicated that the developed repair constructs, including the bioactive components, were non-cytotoxic (viability > 70%). Additionally, a direct co-culture of equine tenocytes and MSCs in the presence of the construct resulted in an increased production of both collagen and non-collagenous proteins, which play an important role in the tendon healing process. In conclusion, the developed repair construct design combining a mechanical and biological approach shows great potential for application in flexor tendon repair. In future experiments, the repair constructs will be evaluated in an in vivo model to confirm these promising findings.

## Supplementary information

Supplementary Information
