# Supplementary material for: Design of an electrospun tubular construct combining a mechanical and biological approach to improve tendon repair
Source: J Mater Sci Mater Med. 2022 May 31;33(6):51. doi: 10.1007/s10856-022-06673-4 (PMC9156498; doi:10.1007/s10856-022-06673-4)
Supplement: Supplementary file 1 — Supplementary Information [file 10856_2022_6673_MOESM1_ESM.docx]

Supplementary Information

Design of an electrospun tubular construct combining a mechanical and biological approach for improved tendon repair

N. Pien*^a,b^*, Y. Van de Maele*^a^*, L. Parmentier*^a^*, M. Meeremans*^c^*, A. Mignon*^d^*, C. De Schauwer*^c^*, I. Peeters*^e^*, L. De Wilde*^e^*, A. Martens^f^, D. Mantovani*^b^*, S. Van Vlierberghe*^a^*, P. Dubruel*^a,*^*

*^a^ Polymer Chemistry & Biomaterials Research Group, Centre of Macromolecular Chemistry (CMaC), Ghent University, Krijgslaan 281 S4-bis, 9000 Ghent, Belgium*

*^b^ Laboratory for Biomaterials and Bioengineering, Department of Min-Met-Materials Engineering & Regenerative Medicine, CHU de Quebec Research Center, Laval University, 2325 Rue de l'Universite, Quebec G1V 0A6, Canada*

*^c^ Faculty of Veterinary Medicine, Department of Translational Physiology, Infectiology and Public Health, Ghent University, Salisburylaan 133, 9280 Merelbeke, Belgium*

*^d^ Smart Polymeric Biomaterials, Surface and Interface Engineered Materials, KU Leuven, Andreas Vesaliusstraat 13 - box 2600, 3000 Leuven, Belgium*

*^e^ Faculty of Medicine and Health Sciences, Department of Human Structure and Repair, Ghent University Hospital, C. Heymanslaan 10, ingang 46, 9000 Gent, Belgium*

*^f^ Faculty of Veterinary Medicine, Department of Large Animal Surgery, Anaesthesia and Orthopaedics, Ghent University, Salisburylaan 133, 9280 Merelbeke, Belgium*


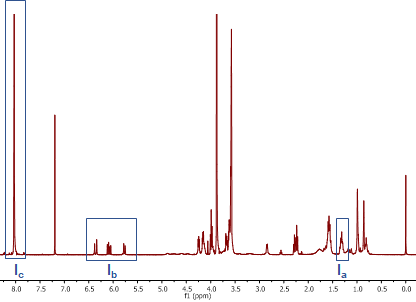


Figure S1. Molar mass determination of AUP 530 through ^1^H-NMR. Related peaks, marked by a blue box, are I_a_ (I_δ_=1.4ppm: integral of signal of the protons from the repeating unit of CL), I_b_ (I_δ_=5.83-6.40 ppm; sum integrals of the signal of the protons in acrylates of AUP 530) and I_c_.(I_δ_=8.00ppm: integral of the signal of the protons from the aromatic ring in DMT).


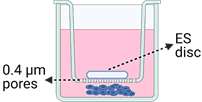


Figure S2. Visualisation of a transwell whereby an ES disc is positioned onto a transparent insert with a pore size of 0.4 µm. Cells, immersed in medium, were located at the bottom of the well. [Created in BioRender.com]


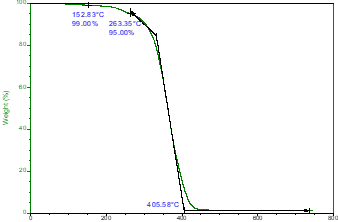

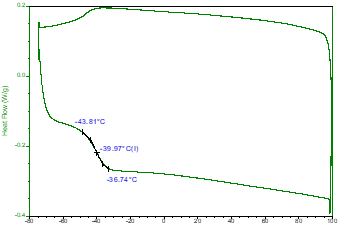


Figure S3. TGA (top) and DSC (bottom) analysis of the synthesized acrylate-endcapped urethane-based (AUP) polymer with a PCL-backbone of 530 g·mol^-1^


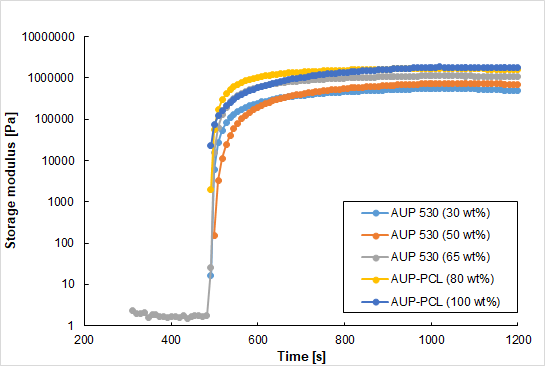


Figure S4. Rheology measurements demonstrating the storage modulus (G’) as a function of time for several AUP 530 concentrations (i.e. 30 wt% and 100 wt%) indicated by different colours. Irradiation of samples with UV-light occurred between 500 and 1000 seconds.

Table S1. Overview of the measured storage moduli and viscosity for various AUP concentrations using rheology.

| AUP concentration | Storage modulus (kPa) | Viscosity (Pa∙s) |
| --- | --- | --- |
| **30 wt%** | 501 ± 11 | 0.17 ± 0.11 |
| **50 wt%** | 704 ± 34 | 0.44 ± 0.02 |
| **65 wt%** | 1 082 ± 10 | 1.48 ± 0.06 |
| **80 wt%** | 1 607 ± 18 | 4.49 ± 0.79 |
| **100 wt%** | 1 773 ± 29 | 91.70 ± 2.72 |

Figure S5. Metabolic activity of indirect (top) and direct (bottom) in vitro testing using human fibroblast cells (hFBs), by an MTS assay at day 1, 3 and 7. Tissue culture plastic was used as a positive control.


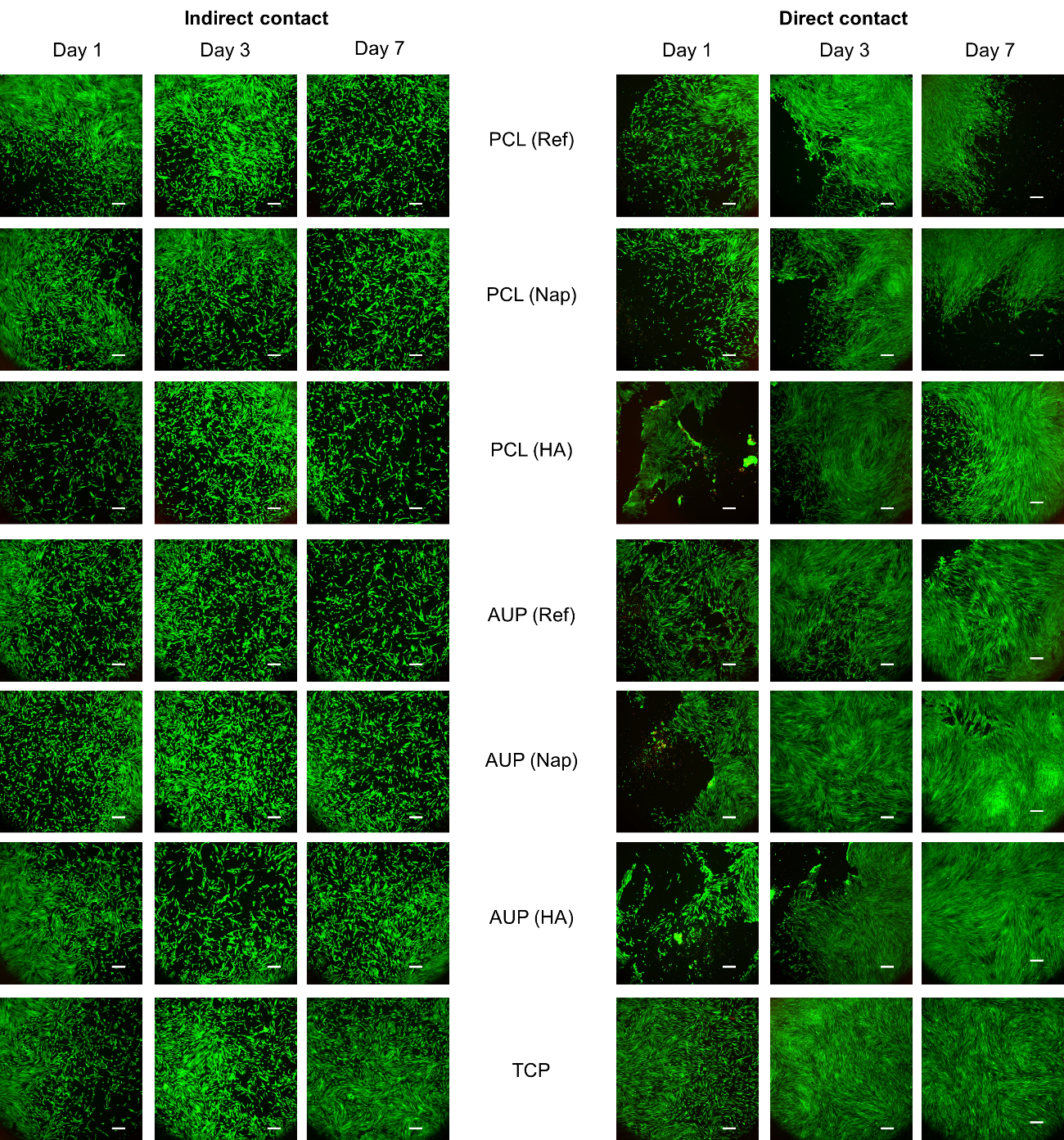


Figure S6. Live/Dead assay of the materials in presence of human fibroblast cells (hFBs) after 1, 3 and 7 days. This was performed in indirect contact (i.e. materials incubated in culture medium) as well as direct contact (i.e. materials placed on top of the cells). Tissue culture plastic (TCP) was used as a positive control.


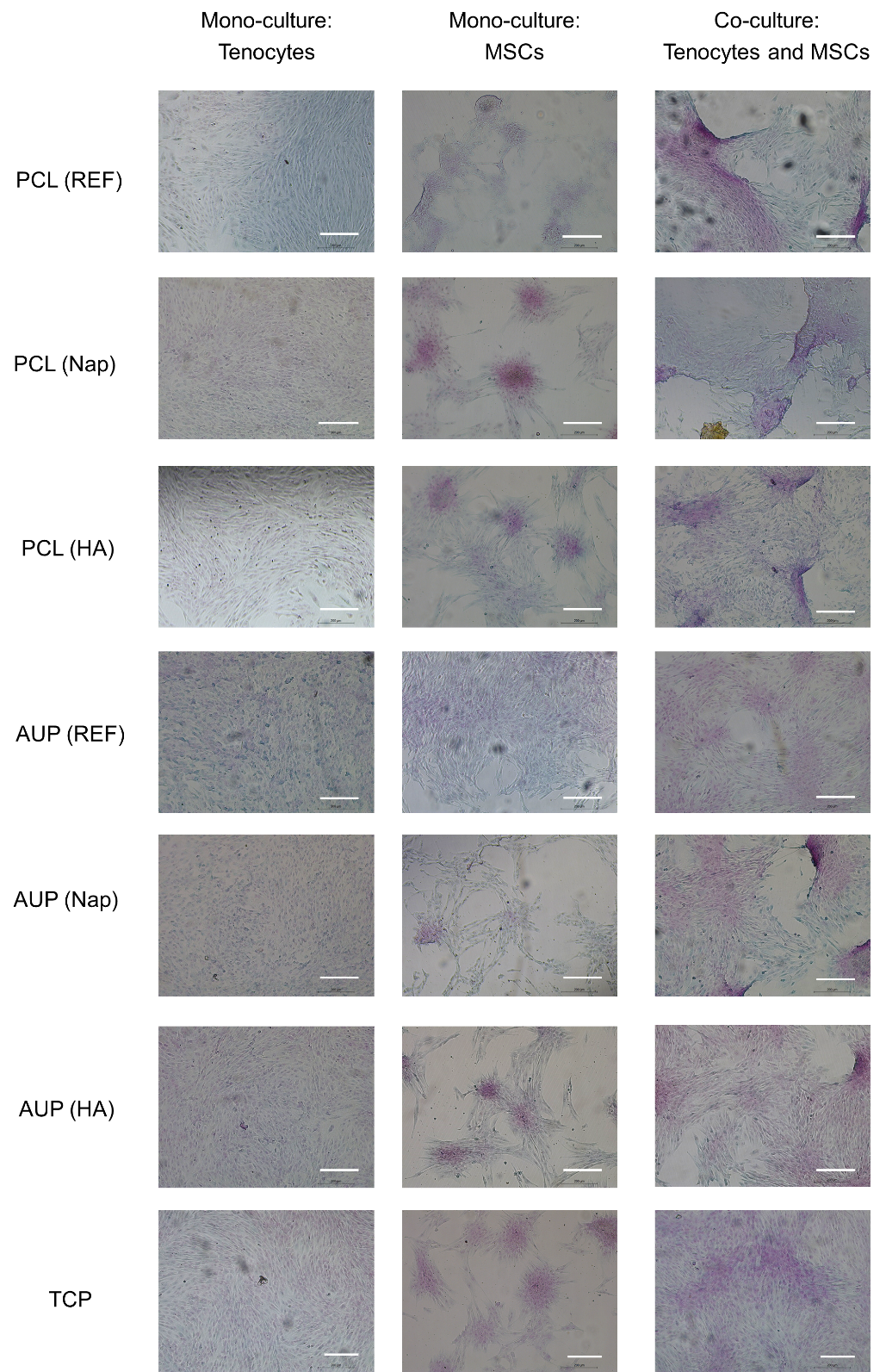


Figure S7. Mono-cultures (tenocytes or MSCs) and co-culture (tenocytes and MSCs) stained with Sirius Red/Fast Green to quantify production of total collagen and non-collagenous proteins. The scale bar indicates 200 µm. Tissue culture plastic (TCP) was used as a positive control.
